# Community-Based Participatory Research on Art Engagement for People with Dementia in Japan

**DOI:** 10.1093/geroni/igaf122.3241

**Published:** 2025-12-31

**Authors:** Mika Sugiyama, Ayako Edahiro, Unno Takako, Furuta Akiko, Fumiko Miyamae

**Affiliations:** Tokyo Metropolitan Geriatric Hospital and Institute of Gerontology, Itabashi City, Tokyo, Japan; Tokyo Metropolitan Geriatric Hospital and Institute of Gerontology, Itabashi City, Tokyo, Japan; Tokyo Metropolitan Geriatric Hospital and Institute of Gerontology, Itabashi City, Tokyo, Japan; The Juntendo Tokyo Koto Geriatric Medical Center, Koutou City, Tokyo, Japan; Tokyo Metropolitan Geriatric Hospital and Institute of Gerontology, Itabashi City, Tokyo, Japan

## Abstract

In Japan, Dementia Friendly Communities promote inclusion and social participation for people with dementia. However, they often face difficulties in engaging in social and cultural activities. This study, part of a community-based participatory research project in Chiyoda City, Tokyo, examined whether participatory art, implemented as a community activity rather than in hospitals or care facilities, could serve as a local resource for post-diagnosis support. The program consisted of an 80-minute pastel and colored pencil participatory art activity, followed by a 40-minute peer support meeting. Six participants with Mild Cognitive Impairment (MCI) or dementia participated, with MMSE-J scores ranging from 14 to 30 (mean 24.7 ± 5.5) and ages from 59 to 97 years. Participants were primarily referred by municipal staff, with some introduced by family. Conducted four times over two months in a community setting, the program had a 91.6% attendance rate. A semi-structured group interview was conducted in the final session, and thematic analysis was performed. Thematic analysis revealed that participatory art activities provided “fun and enjoyment,” opportunities to “try new things,” and the “joy of self-expression.” Many participants noted that they had not engaged with art on their own and expressed a decrease in opportunities to experience art in daily life. Many hoped for continued participation in participatory art activities. These findings suggest that participatory art, when integrated into community activities, can be a valuable resource for people with dementia and MCI, fostering social participation and self-expression. Future research should explore sustainable models for community-based dementia-friendly initiatives.

